# Supplementary material for: Genotypes and phylogenetic analysis of adenovirus in children with respiratory infection in Buenos Aires, Argentina (2000–2018)
Source: PLoS One. 2021 Mar 8;16(3):e0248191. doi: 10.1371/journal.pone.0248191 (PMC7939361; doi:10.1371/journal.pone.0248191)
Supplement: S1 Fig — (PDF) [file pone.0248191.s001.pdf]

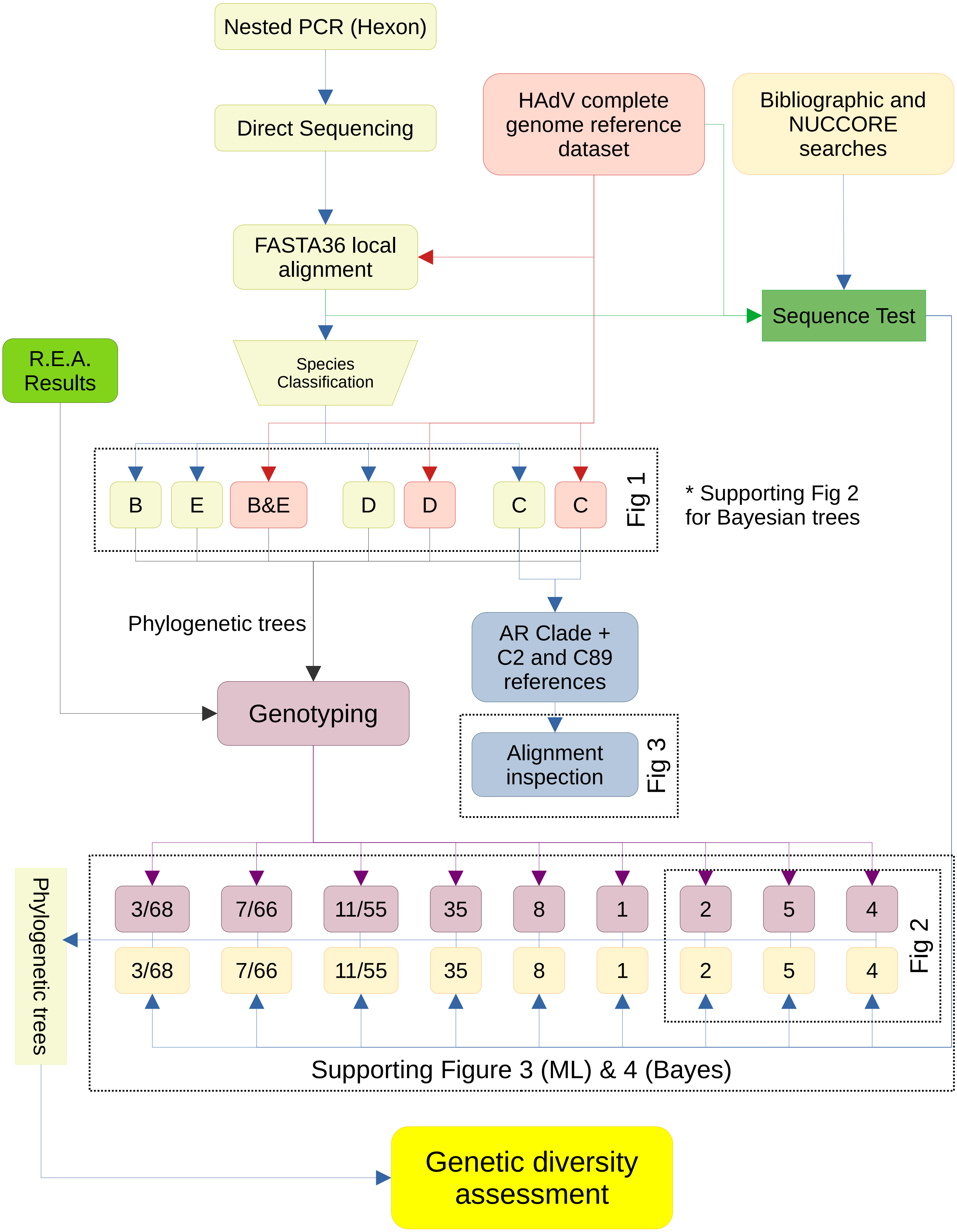

S1 Fig. Data acquisition and analysis. The main steps of how data was obtained (sequenced, dataset construction or database query), how the datasets were created for the different phylogenetic reconstructions, and how other information, as alignment statistics and R.E.A. results were used to interpret the resulting trees.
