# Supplementary material for: Genotypes and phylogenetic analysis of adenovirus in children with respiratory infection in Buenos Aires, Argentina (2000–2018)
Source: PLoS One. 2021 Mar 8;16(3):e0248191. doi: 10.1371/journal.pone.0248191 (PMC7939361; doi:10.1371/journal.pone.0248191)
Supplement: S2 Fig — (PDF) [file pone.0248191.s002.pdf]

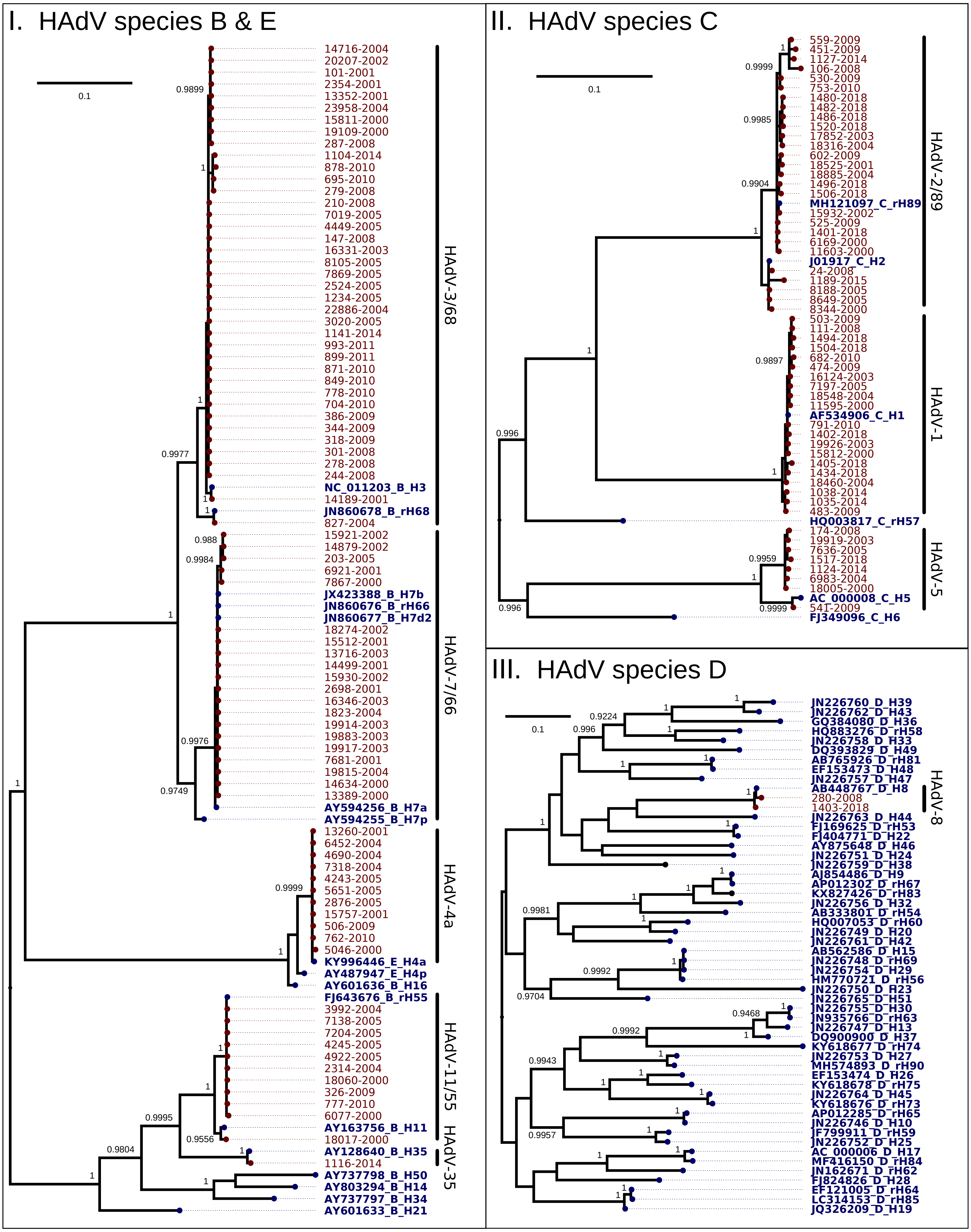

S2 Fig. HAdV strains from children with acute respiratory infection in Buenos Aires, Argentina -2000 to 2018 . Buenos Aires samples are in red, identified by the laboratory number followed by the isolation year. GenBank accession numbers of all available reference sequences (blue) are shown. Phylogenetic trees obtained by Bayesian methodology based on the HAdV partial hexon gene (HVR 1-6) by HAdV species. For each dataset the best fit model was used: I. TPMuf+Γ (nst=6 rates=gamma), II. TIM2+ Γ+I (nst=6 rates=invgamma), III. TIM2+ Γ+I (nst=6 rates=invgamma). As branch support the posterior clade probability is shown near (above o below) the node. Only posterior values higher than 0.95 were shown. The topologies are equal to those obtained by maximum likelihood. Only minor differences in brach lenghts were found.
