# Supplementary material for: Genotypes and phylogenetic analysis of adenovirus in children with respiratory infection in Buenos Aires, Argentina (2000–2018)
Source: PLoS One. 2021 Mar 8;16(3):e0248191. doi: 10.1371/journal.pone.0248191 (PMC7939361; doi:10.1371/journal.pone.0248191)
Supplement: S3 Fig — (DOCX) [file pone.0248191.s003.docx]

S3 Fig.

# HAdV Genetic Diversity Assessment

Tree topologies obtained by Maximum Likelihood methodology. Branch supports obtained by standard non-parametrical bootstrapping method. Red: Buenos Aires sequences belonging to this study and named including the identification number followed by the year of collection. Blue: Genotype reference sequence. Sequences were named including its GenBank Accession Number following the country where the sample was collected in two letters ISO code and the year of collection in a four digit code. Missing collection countries or dates are marked with “??”. Nucleotide substitution models: HKY=Hasegawa, Kishino and Yano 1993; F81=Felsenstein 1981; K3P=Kimura 3 parameters; +I or +Γ = substitution rate heterogeneity was modeled as proportion of invariable sites or with a gamma distribution, respectively.

### HAdV-B3 and B68


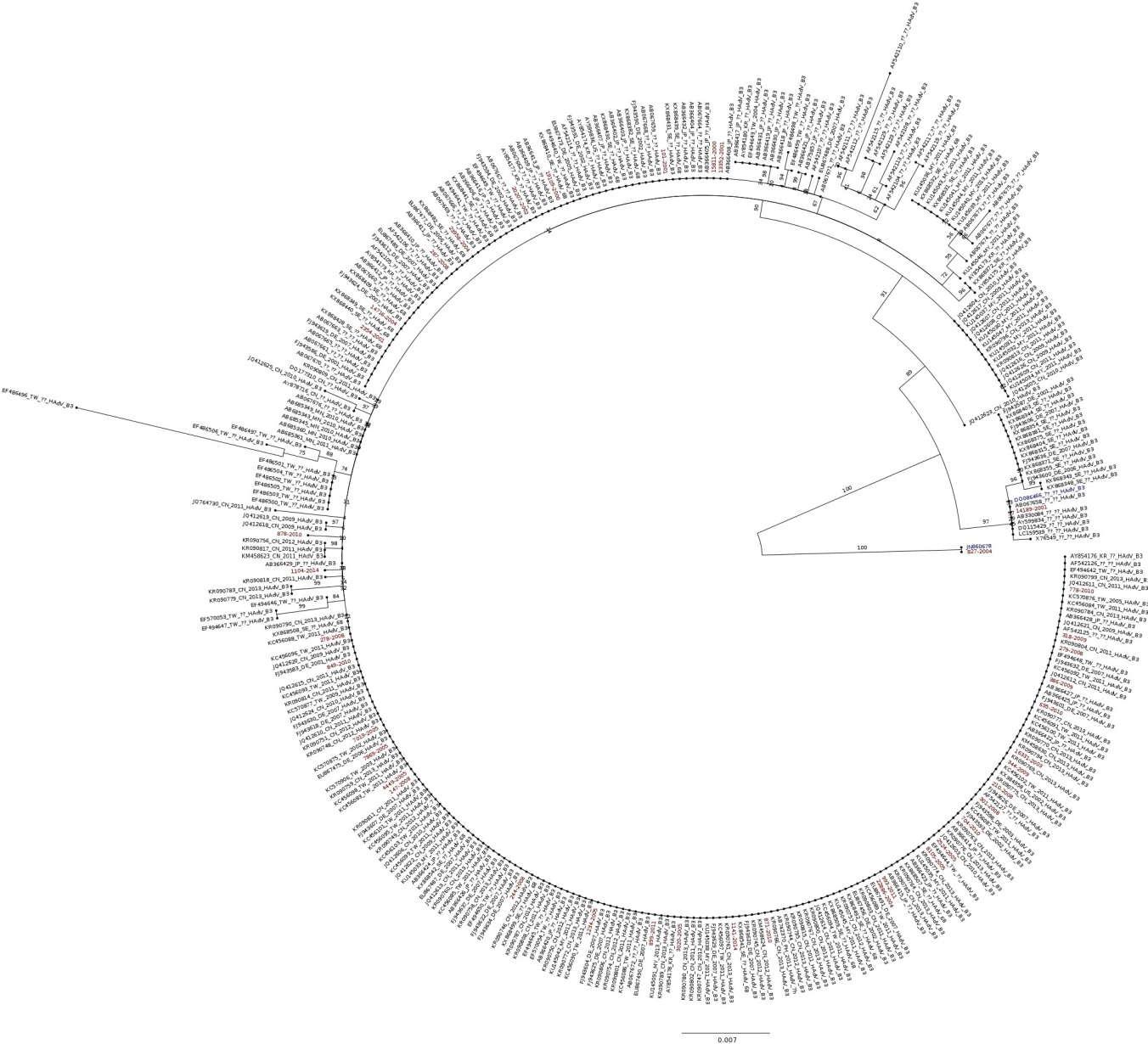


Dataset for genotype 3 and 68 included 350 sequences. The best nucleotide substitution model according to BIC was HKY+Γ. The analysis showed two clusters: a large one containing 326 sequences with worldwide distribution (China, Japan, Korea, Malaysia, Mongolia, Philippines, Germany, Sweden and USA) including 37/39 of our strains, a small one containing 22 sequences from Europe (Germany and Sweden) and 1 of our strains. A separate group, including genotype 68 reference strain and one of our strains, showed a longer phylogenetic distance to the large and the small clusters.

### HAdV-B7 and B66


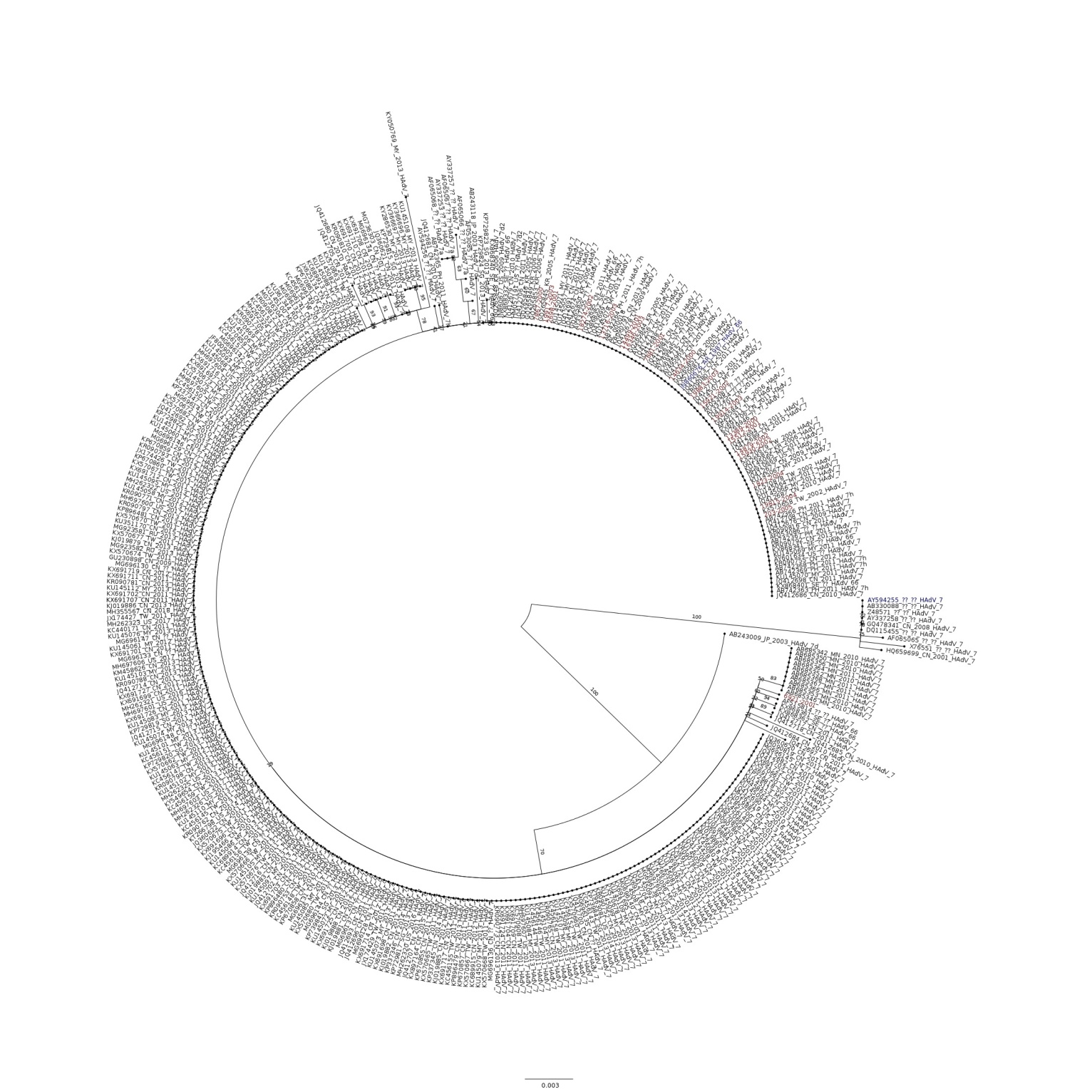


Dataset for genotypes 7 and 66 included 394 sequences. The best nucleotide substitution model according to BIC was HKY. The phylogenetic tree showed two clusters. A big one included 385 sequences from China, Japan, Korea, Malaysia, Mongolia, Philippines, Germany, Sweden, Russia, Argentina and USA, along with references strains for types 7a (vaccine strain), 7d, 7d2 and 66 (previously known as 7h). All our strains grouped in this large cluster. The remaining 9 sequences formed a small cluster and were related to type 7p (Gomen strain).

### HAdV-B11 and B55


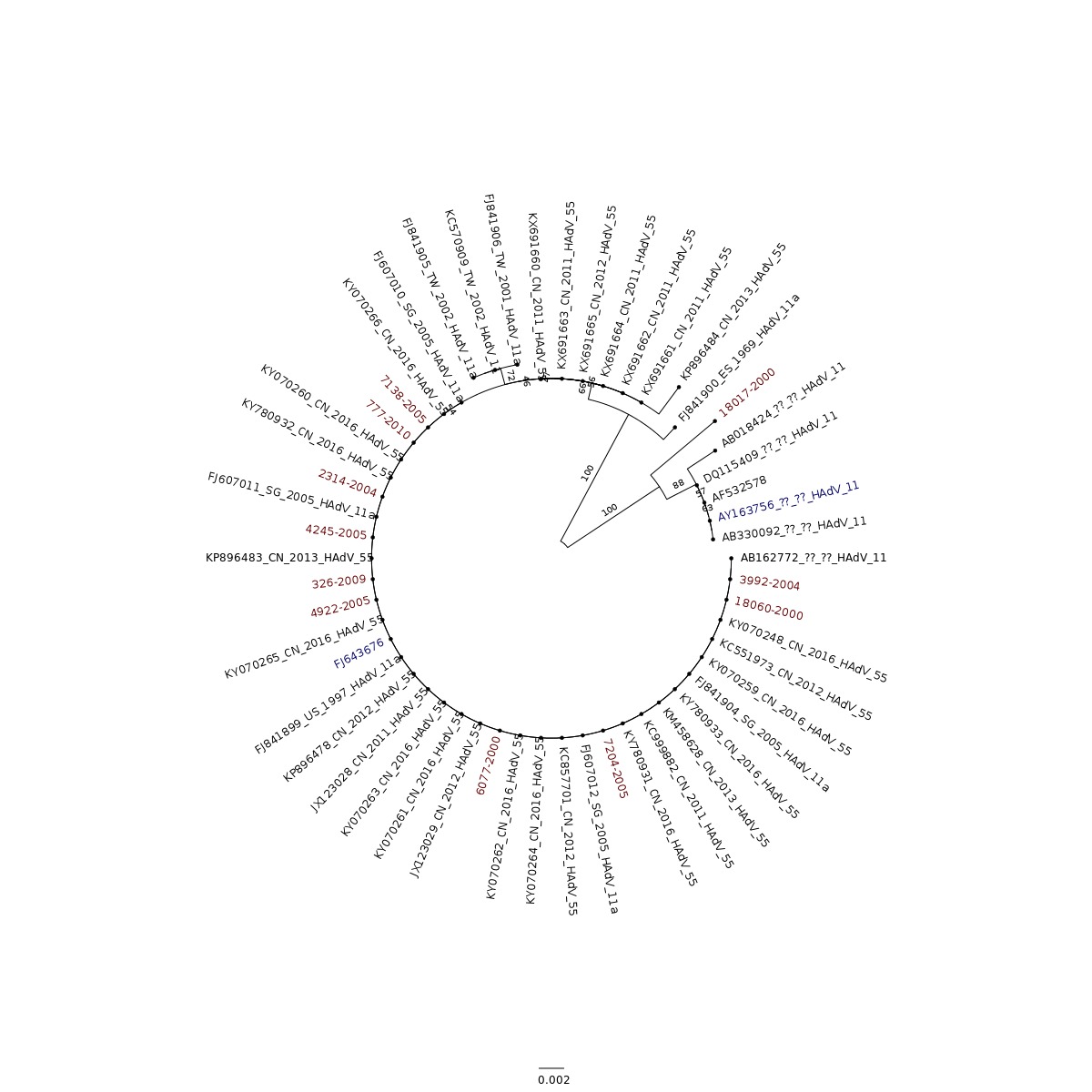


Dataset for genotypes 11 and 55 included 54 sequences. The best nucleotide substitution model according to BIC was HKY. The phylogenetic analysis showed two clusters. A large one, which contained the reference genotype 55, 10 sequences from our study and 37 highly similar sequences from China, Singapore, Spain and the USA. The remaining 6 sequences formed a small cluster with reference genotype 11 and one strain from our study. This last strain was the most divergent sequence within this small cluster.

### HAdV-B35


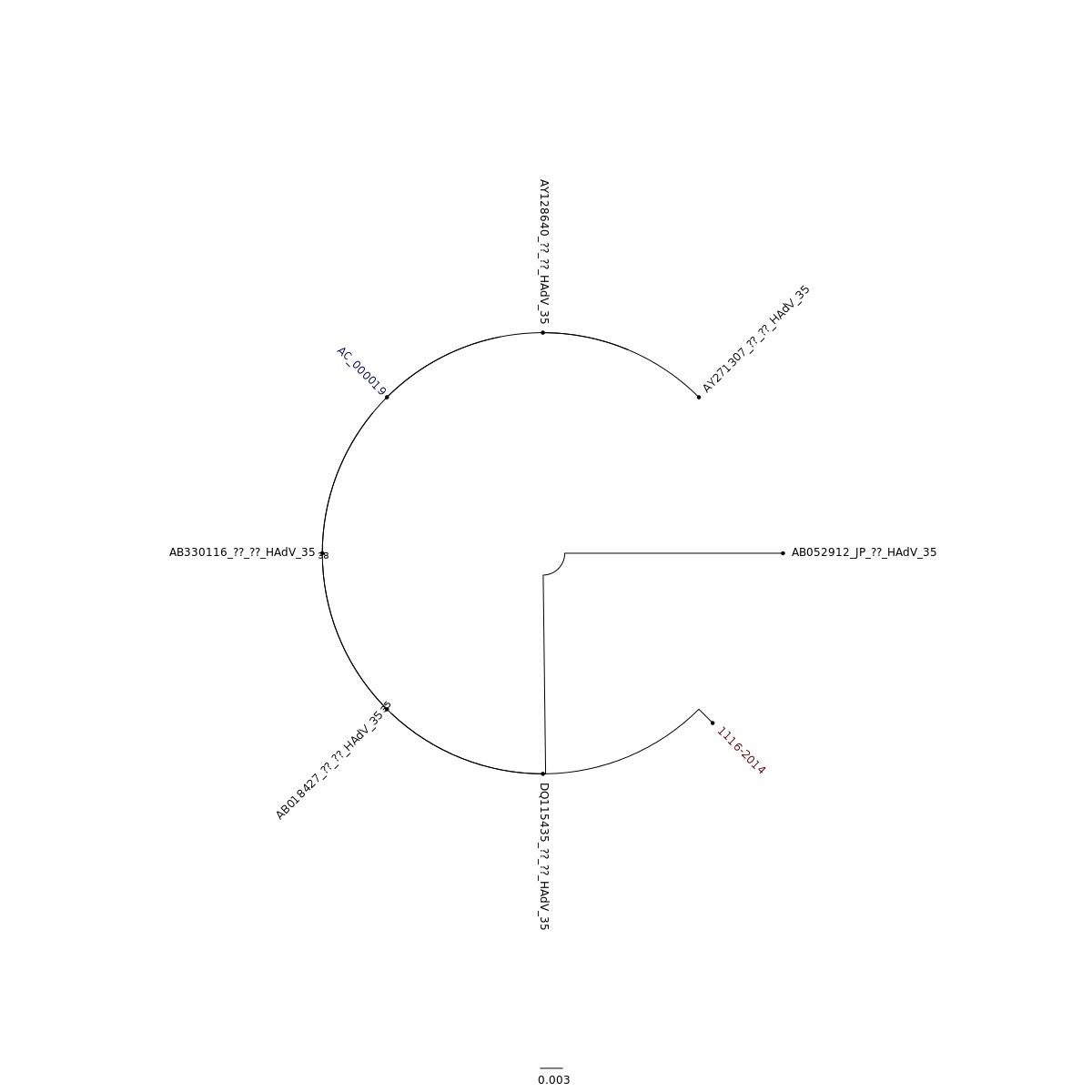


Only 8 sequences composed the genotype 35 dataset. The best nucleotide substitution model according to BIC was HKY. Six of them were identical and had no information about their collection site. The sample from our study belonging to this genotype was very similar to these six sequences. The eighth sequence was phylogenetically more distant and was collected in Japan.

### HAdV-C1


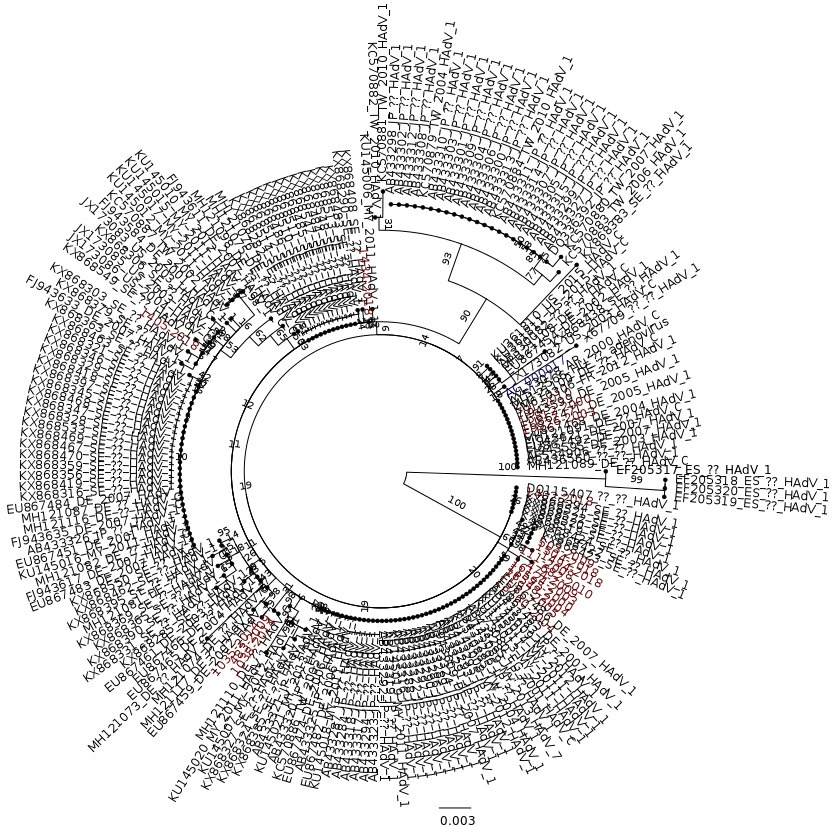


Dataset for genotype 1 contained 209 sequences. The best nucleotide substitution model according to BIC was HKY+I. The phylogenetic tree showed a main group of sequences that were identical or very similar to the reference genome. This basal group included strains from China, Japan, Malaysia, France, Germany, Hungary, Sweden, Egypt, Argentina and USA. Most of our strains (17 out of 20) lied within this main group. Six supported clusters (with 3 or more sequences) stem from this basal group. Four of these clusters were phylogenetically close to the main group, two including sequences from different places (one from Germany, Sweden, USA and our strain, and the other from Germany, Sweden and Malaysia) and the other two, included sequences from a single location (one from Malaysia and the other 3 sequences from our strains). The remaining two clusters were phylogenetically more distant to the main group and included sequences from a single geographic area, one with 4 sequences from Spain and the other with 27 sequences from Japan and Taiwan.

### HAdV-C2


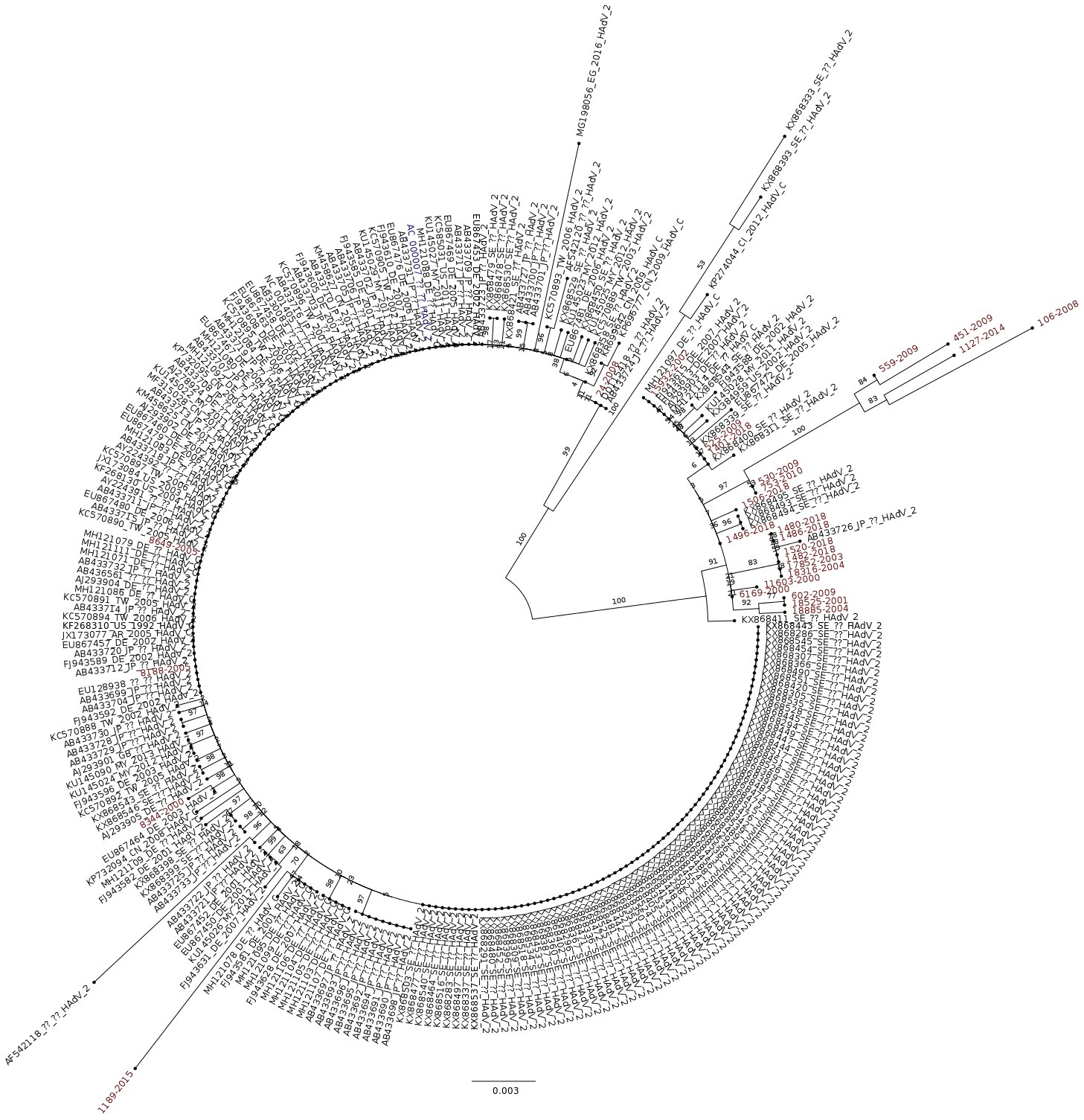


Dataset for genotype 2 included 268 sequences. The best nucleotide substitution model according to BIC was HKY+I. The phylogenetic analysis showed a big cluster of 224 sequences that were identical or very similar to the reference HAdV-C2 (AC_00007). These strains were collected from China, Japan, Malaysia, Germany, Sweden, the United Kingdom, Egypt, Argentina and USA. Only 5 among 27 of our strains were found in this big cluster. Most sequences were intermingled and five small supported groups were observed, each of them was location-specific. A small cluster including 44 sequences was divided into two sister groups: one with 3 sequences (Sweden and Côte d'Ivoire) and the other group with 22 of our strains and 19 sequences from Japan, Malaysia, Germany and Sweden. Within our 22 strains, 6 formed a supported cluster.

### HAdV-C5


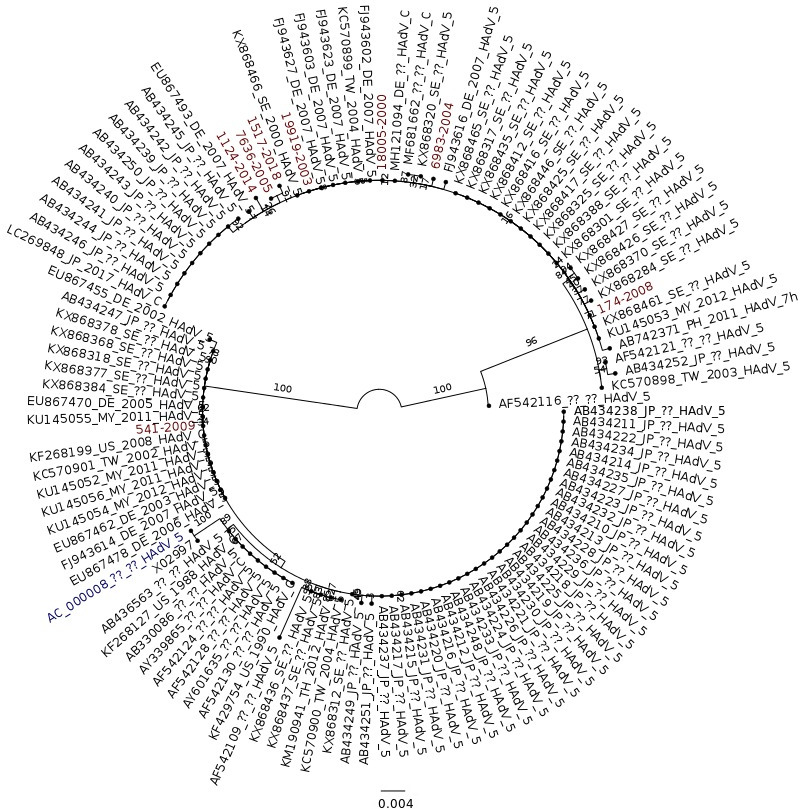


Dataset for genotype 5 included 117 sequences. The best nucleotide substitution model according to BIC was HKY+I. The phylogenetic analysis showed two clusters with 67 and 50 sequences, respectively. The first cluster contained sequences from China, Japan, Malaysia, Thailand, Germany, Sweden, USA and 1 of our strains. The second cluster contained sequences from China, Japan, Malaysia, Philippines, Germany, Sweden and 7 of our strains.

### HAdV-D8


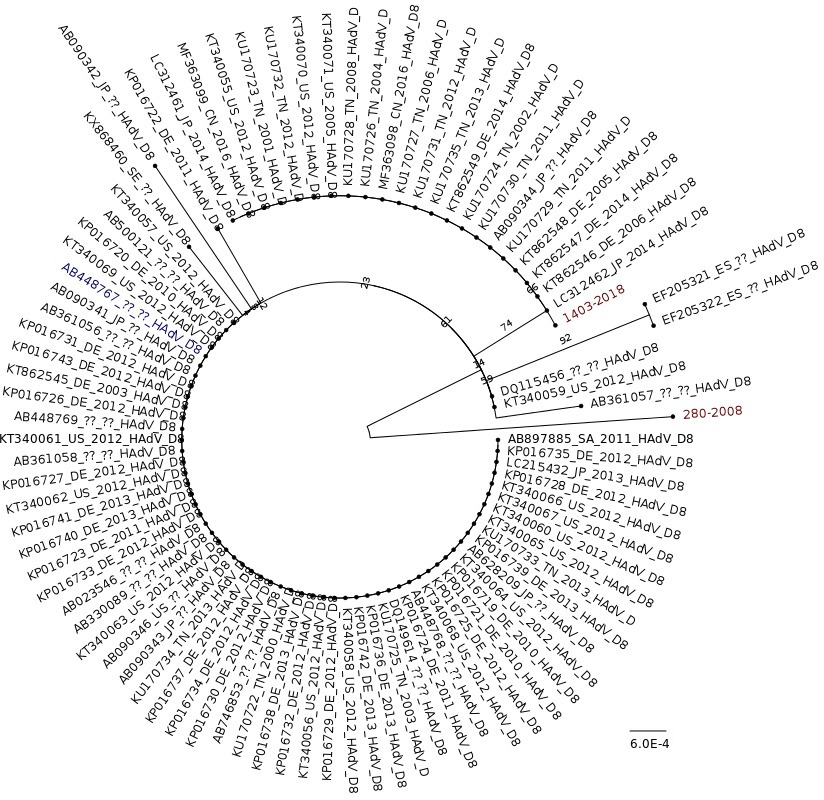


Dataset for genotype 8 included 90 sequences. The best nucleotide substitution model according to BIC was F81. When compared with the reference genome, 60 sequences were identical, 22 sequences showed only one nucleotide difference (synonymous substitution) and 4 sequences had 1 or 2 nucleotide differences in other positions. This dataset included sequences from Tunisia, Japan, Saudi Arabia, Germany, Spain, Sweden and USA. One of the two sequences of our strains shared the above mentioned synonymous substitution, but the other was the more divergent sequence in the dataset.

### HAdV-E4


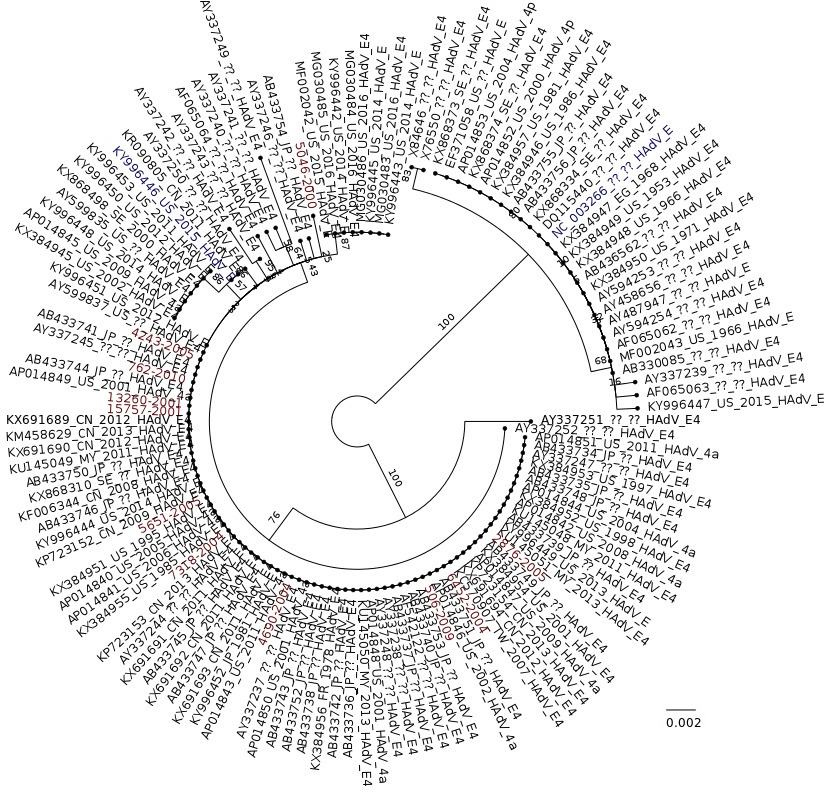


Dataset for genotype 4 included 134 sequences. The best nucleotide substitution model according to BIC was K3P+I. The phylogenetic analysis showed two clusters. A large one related to genome type 4a, included 105 sequences from USA, China, Japan, Malaysia, Sweden and all 11 from our strains. A small cluster related to genome type 4p, included 29 sequences from USA, Egypt, Japan and Sweden.
