# Supplementary material for: Genotypes and phylogenetic analysis of adenovirus in children with respiratory infection in Buenos Aires, Argentina (2000–2018)
Source: PLoS One. 2021 Mar 8;16(3):e0248191. doi: 10.1371/journal.pone.0248191 (PMC7939361; doi:10.1371/journal.pone.0248191)
Supplement: Attachment — Submitted filename: PONE-D-20-36189_review_14-12-2020.docx [file pone.0248191.s006.docx]

The article entitled “Genotypes And Phylogenetic Analysis Of Adenovirus In Children With Respiratory Infection In Buenos Aires, Argentina (2000 - 2018)” by Débora N. Marcone et al. is a cross-sectional study using HAdV positive respiratory samples from children with ARI, in Buenos Aires. The topic of the article is interesting and medically important. The manuscript is well written. My comments to the authors are as follows:

**Abstract:** OK.

**Introduction:**

Line 44: Describe the common adenoviral and occasional adenoviral disease separately.

**Methods:**

It will be well understood for the reader if the whole method is shown with the help of a flowchart.

Line 111, 112: Explain the rationale of direct PCR-sequencing of L1 (HVR1-6) of the hexon.

Line: 135-37: REA enzyme profile can be added as a supplementary file if available.

**Result:** OK

**Discussion and conclusion:** OK
